# Network Coding on Heterogeneous Multi-Core Processors for Wireless Sensor Networks

**DOI:** 10.3390/s110807908

**Published:** 2011-08-11

**Authors:** Deokho Kim, Karam Park, Won W. Ro

**Affiliations:** 1 The School of Electrical and Electronic Engineering, Yonsei University, Seoul 120-749, Korea; E-Mail: nautes87@yonsei.ac.kr; 2 Mobile Communications, Samsung Electronics, Suwon 443-373, Korea; E-Mail: karam.park@samsung.com

**Keywords:** network coding, sensor nodes, parallel algorithms, heterogeneous multi-core processors

## Abstract

While network coding is well known for its efficiency and usefulness in wireless sensor networks, the excessive costs associated with decoding computation and complexity still hinder its adoption into practical use. On the other hand, high-performance microprocessors with heterogeneous multi-cores would be used as processing nodes of the wireless sensor networks in the near future. To this end, this paper introduces an efficient network coding algorithm developed for the heterogenous multi-core processors. The proposed idea is fully tested on one of the currently available heterogeneous multi-core processors referred to as the Cell Broadband Engine.

## 1. Introduction

Network coding is a new coding technique first proposed by Ahlswede *et al*. to enhance network throughput and effectiveness on multi-nodal environments [1] such as wireless sensor networks (WSN). A new paradigm has emerged for computer network systems enabled by network coding; advances in network coding techniques have influenced information and coding theory, computer network performance, and wired/wireless communication systems. In addition, network coding lends itself particularly well to multicasting, enhancing the effectiveness of multicasting compared to traditional coding approaches.

In fact, use of network coding techniques to various real world applications has been introduced [2,3]. Further more, network coding has the potential to deliver a number of benefits in various domains such as wireless networks, sensor networks, network security, peer-to-peer (P2P), and on-demand video streaming service [2,4–16]. In wireless network systems, the network coding can increase transmission efficiency at routers by forwarding the coded packets as a passive acknowledgement [11] and can increase performance of ad-hoc networks as well as save the energy with many to many broadcast environment [12,13]. In addition, the coded packets which are on the fly cannot be decoded until the sufficient number of packets are collected, thus network coding can simplify implementation of secure network as well [14,15]. In practical approach, Liu *et al*. analyze the performance of network coding on real-world commercial systems with 200 GBytes of real-world traces which had been collected during Summer Olympic Games in 2008 [16].

While network coding has several advantages and is a promising technique for the future of network systems, one crucial drawback is the associated volume of computational overhead, which may hinder its adoption in practical use. Network coding requires encoding the data before it is sent and decoding it after it is received. However, the decoding algorithm has *O*(*n*^3^) computational complexity, using a variant of Gaussian elimination where *n* is the size of a coefficient vector. The computation overhead associated with the decoding operation is very costly, especially with the low computing environment such as wireless sensor networks. As a result, the benefits of the network coding technique may be canceled out by the long decoding delay.

On the other hand, multi-core processors have recently become widespread and can be found in a variety of systems [17], from high performance servers to special purpose wireless sensor networks [18,19]. In fact, the current research on sensor networks mainly uses a light-weighted processing node as a sensor node. However, we also expect that the future WSN systems would require more computing power, especially for the multimedia sensors. Therefore, the multi-core processor would be a possible choice for the sensor node. Especially, a prototype of multi-core platform as a sensor node is introduced in the previous research [18]. This paper is based on the expectation that the future WSN would popularly use multi-core processors and require parallelized random linear network coding. In addition, using advanced microprocessor features such as the multimedia extension in WSN is investigated in the previous literature as well [20]. In fact, processor development has resulted in a progressively increasing number of cores in a single chip. There are two kinds of multi-core processor design paradigm; one group integrates homogeneous multiple cores on a single chip whereas the other group incorporates heterogenous cores.

In this paper, we present a parallel algorithm of network coding for heterogeneous multi-core processors especially targeting to utilize the technique in WSN. We select the already available heterogeneous platform, the Cell BE, as a prototype of heterogeneous multi-core processors and adjust the workload distribution on each core for efficient network coding. The Cell BE is a heterogeneous multi-core processor designed to provide both generality and intensive computing power with the single instruction multiple data (SIMD) paradigm. Therefore, the design of Cell BE lends itself well to the adoption of SIMD which can be efficiently utilized in wireless multimedia sensor networks [20]. Indeed, GPU also can be chosen as a high performance computing device for wireless sensor networks. However, we concern that using GPU requires additional general purpose processor support. This might introduce additional hardware and software overhead.

In fact, using the Cell BE processor in sensor nodes may not be so desirable due to the size and power consumption. However, the main target of this paper is to show the efficient parallel algorithms of the network coding on heterogeneous processors and demonstrate the possible advantages and feasibility of the algorithm. We formulate an appropriate load balancing method to achieve this, which is based on the concept of divisible load theory (DLT), which was initially introduced by Bharadwaj *et al*. and Drozdowski in the context of distributed and cluster systems [21–23]. In addition, we consider three different approaches incorporating parallelized decoding across the multiple heterogeneous cores, employing Galois field computation methods.

Via real machine experiments, we demonstrate that the proposed technique delivers improvements in decoding speed. With proper load balancing, we achieve a maximum speed-up of 2.15, compared to the performance results without load balancing. In addition, we compare our idea to the results obtained in two homogenous multi-core processors which provide competitive computing power. Compared to the Intel quad-core system, our approach achieves a maximum speed increase of 2.19, with 1 MB of data and a coefficient matrix of size 64 × 64. When we compare our performance to that of an AMD processor, we observe a maximum speed-up of 3.12 for 128 KB of data and a coefficient matrix of size 64 × 64.

The rest of this paper is organized as follows. We describe the network coding theory and brief overview of the Cell BE architecture in Section 2. Then, we propose parallelized network coding implementations for use on the Cell BE, as well as an extension to the SIMD instruction set in Section 3. In Section 4, experimental performance results are presented and analyzed. In Section 5, related works are explained. Finally, we conclude the paper in Section 6.

## 2. Background

In this section, we will first introduce the overview of the Cell BE. In addition, some necessary knowledge on the concept of network coding will be presented.

### 2.1. Overview of the Cell BE

**Figure 1.**
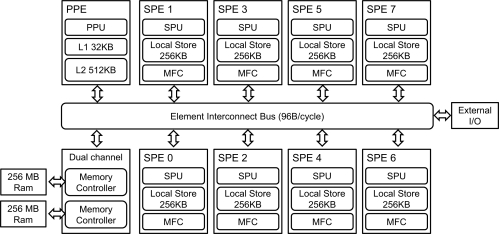
The block diagram of the Cell BE architecture.

The Cell Broadband Engine (Cell BE) is a heterogeneous multiprocessor that was developed by Sony, IBM, and Toshiba in 2000. Although it has been long time from the first release of the Cell BE, it has 256 GFLOPS (Giga Floating Operation Per Second). It still provides good performance as a single chip processor compared to one of today’s high-performance commercial processors, Intel Core i7 series (Intel Core i7 975 has theoretical performance 221.44 GFLOPS) [24]. In addition, the Cell BE is appropriate to show heterogeneous program models. The Cell BE consists of one Power 64 architecture processor, referred to as a *Power Processor Element* (PPE), and eight co-processors, referred to as *Synergistic Processor Elements* (SPEs). The Cell BE also includes Directed Memory Access (DMA) controller and high bandwidth data bus, referred to as an Element Interconnection Bus (EIB). These various components are presented in Figure 1. The Cell BE processor incorporates a Single Instruction Multiple Data (SIMD) execution unit, high power and area efficiency, large memory bandwidth, a large bandwidth on-chip coherent bus, and a high-bandwidth flexible I/O [25].

The PPE is a dual-threaded, dual-in-order issue 64 bits Power-architecture processor. It has a 32 KB instruction cache and a 32 KB data cache, as well as a 512 KB L2 cache. In addition, the PPE has an *AltiVec* vector extension unit and floating point and integer SIMD instruction set.

The SPEs are composed of a Synergistic Processor Unit (SPU), a 256 KB local store, and a Memory Flow Control (MFC). The execution performance of the SPEs affects much of the overall computational performance of the Cell BE. The SPU contains a 128 bit wide dual-issue SIMD unit fully pipelined to all precisions, with the exception of the double precision vector unit. The SPE can access main storage with an effective address (EA) translation by MFC and asynchronously transfer data to local storage, which has both narrow (128 bits) and wide (128 bytes) features.

The Element Interconnect Bus (EIB) is a coherent bus that can transfer up to 96 bytes/s. It consists of four 16 bytes rings, each of which is only capable of unidirectional data transfer, clockwise or counter-clockwise, each ring supporting up to three simultaneous data transfers. The Cell BE employs dual channel *Rambus* XDR DRAM, which is capable of transferring 12.8 GB/s per 32 bits memory channel. It is therefore capable of supporting total bandwidth of 25.6 GB/s [26].

### 2.2. Benefit of Using Network Coding

**Figure 2.**
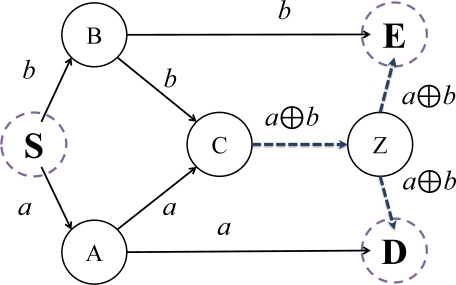
Advantage of using network coding.

We will introduce the principles and advantages of using network coding in this subsection. Figure 2 presents a simple example of communication networks, which is represented as a directed graph [1].

Each directed edge represents a pathway for information transfer. Node *S* represents source and the nodes *D* and *E* are destinations. The other nodes are intermediaries, routing information to the destination nodes. If we assume that each link is limited in bandwidth one bit per unit time, in a traditional routing protocol, we are incapable of attaining higher throughput than the given limit. However, using network coding, we can achieve better throughput in excess of this limit.

Let us assume that we have generated data bits *a* and *b* from source node *S*, and that we wish to route the data to destination nodes *D* and *E*. Data bit *a* is transported via path *S-A-C*, *S-A-D* and data bit *b* via *S-B-C*, *S-B-E*.

At the edge spanning nodes *C* and *Z*, constrained by our bandwidth limitation, we can only transport one of either *a* or *b*, per unit time. Suppose that we send *a* along the edge between nodes *C* and *Z*. In this case, node *D* could not receive *b* and would only be capable of receiving *a* twice, from *A* and *Z*. In addition, if we send *b* at the same time, node *E* would face the same problem. As it is not possible to transfer data bits *a* and *b* to both nodes *D* and *E* simultaneously, routing is inadequate.

When using network coding, we are able to generate new data by first encoding *a* and *b*, and then routing the encoded data through the directed linkage between nodes *C* and *Z*. As a simple example, we use a bitwise *xor* to encode data bits *a* and *b*. The new data is thus encoded as ‘*a xor b*’ and is sent along paths *C-Z-D* and *C-Z-E*, simultaneously. Node *D* would therefore receive data bits *a* and (*a xor b*) from nodes *A* and *Z*, respectively. Further, node *E* would receive both data *b* and (*a xor b*) from nodes *B* and *Z*. Therefore, both nodes *D* and *E* can collect data bits *a* and *b* using the *xor* operation. In conclusion, using a network coding technique allows us to achieve an enhanced multicast throughput of two bits to both nodes, subject to the same base network capacity of *one bit per unit time*.

### 2.3. Random Linear Network Coding

To fully leverage the potential benefits of the network coding technique in a practical system, the encoding and decoding operations must be fast enough (*i.e.*, they must not act as bottlenecks to the transmission process). The execution time of the network coding is primarily dependent upon the coding method used. We employ the random linear coding [27] in our Cell BE implementations, as it is widely used and known to be asymptotically optimal in any network format.

**Figure 3.**
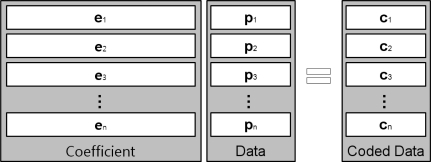
Data encoding at the sending node.

**Figure 4.**
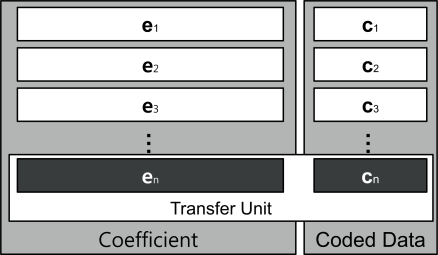
Data received at the receiving node.

A given segment of data, such as a single file, will be divided into a specific number of blocks, referred to as *packets*, prior to being transferred over a network, as shown in Figure 3. In this figure, **p***_k_* represents *k^th^* block and **c***_i_* is a coded data, which is a linear combination of blocks. In other words, $c_{i}=\Sigma_{k=1}^{n}e_{i,k}p_{k}$ where *n* is the number of blocks and the coefficient **e***_i_* is an element vector that is selected at random from a finite field, *F*. The coded data **c***_i_* is combined with the coefficient vector; [*e_i_*_,1_, ..., *e_i_*_,_*_n_*] is stored in the header and broadcast to the destination. A transfer unit, comprised of the coded data and coefficient block, is presented in Figure 4.

While the packets are being routed, the packets are re-encoded within nodes along the pathways to their destinations before being passed to downstream nodes. When a packet arrives at its destination node, it is stored in local memory so the coded data can be decoded and recovered to the original data set [*p*_1_, ..., *p_n_*]^T^. To decode encoded data, the destination node must have all *n* transfer units, with linearly independent coefficient vectors. Suppose a destination node has collected *n* transfer units and that the coefficient vector, original data, and coded data set are represented by $E^{T}=[e_{1}^{T},\ldots ,e_{n}^{T}]$, $C^{T}=[c_{1}^{T},\ldots ,c_{n}^{T}]$, $P^{T}=[p_{1}^{T},\ldots ,p_{n}^{T}]$, respectively, where superscript *T* implies a matrix transpose operation. Since we multiply the matrices with formula *C* = *EP* to encode original data, we can rearrange this to obtain *P* = *E*^−1^*C*, allowing us to recover the original data by multiplying the inverse matrix of *E* with *C*. To perform the decoding operation, the coefficient matrix, E, must be an invertible matrix, thus all coefficient vectors, **e***_i_*, should be linearly independent of each other.

Using a variant of *Gaussian Elimination*, we can obtain matrix *P*. When the destination receives transfer units, it constructs coefficient and coded data matrices, as shown in Figure 4, to prepare for the process of Gaussian elimination. Typical Gaussian elimination or LU decomposition for the purpose of decoding at the destination requires that all *n* transfer units first be collected, before starting the process. However, we can use progressive decoding instead of multiplying by the inverse matrix. With the progressive decoding [28], we do not need to wait until all transfer units to be received. Although all units may not have been received, the decoding process can still be initiated, and continue to progress as each unit is made available. In addition, the progressive network coding can be processed regardless of the arrival order of the coded packet. It is due to the fact that changing of the row order does not affect the decoding results as it uses linearly independent coefficient matrix.

Let *n* represent the number of blocks and *m* represent the block size. The computation complexity of standard Gaussian Elimination is *O*(*n*^3^). However in the decoding process associated with network coding, there is an extra matrix of size *m*, represent by **c***_i_* in Figure 4. Therefore, the computational complexity in network coding is increased to *O*((*n* + *m*) × *n*^2^).

An additional peer within a file swarming system can reduce download delay by *n*, receiving at most *n* block simultaneously. However, the resultant decoding delay, which increases in proportion to *n*^3^, offsets the reduction in download delay, thus the benefit is canceled out. Therefore, in order to achieve some measure of benefit from a large *n*, an efficient, fast decoding implementation is required. That is, we can achieve greater performance gains in larger *n*, if we are able to overcome the computational delay.

### 2.4. Overview of Progressive Network Coding

A variety of decoding methods that employ the random linear network coding technique is based on matrix inversion algorithms [29,30]. Though using the traditional algorithms is a proven method of parallel decoding in network environments, there is an additional cost incurred from network transmission delay. As the system must wait until all packets are received to compose the matrices used in the aforementioned traditional decoding algorithms, this delay is particularly problematic. As such, we can obtain greater performance using progressive decoding in packet switching network environments, which are subject to these transmission delays.

The traditional matrix inversion algorithms require a complete matrix to perform the decoding operation; this results in additional delays due to the waiting period. In contrast, progressive decoding requires only one row of the matrix to proceed with decoding. As such, progressive decoding is more suitable to network environments that are subject to long transmission delays.

The decoding process for traditional matrix inversion algorithms can be expressed with a computational complexity of *O*(*n*^3^), after the last row has arrived. However, with the progressive decoding we can initiate the decoding process when as each row is received. Since we have already finished computation of all prior rows, the most recent row can be processed with complexity of *O*(*n*^2^). In our evaluation, we employs progressive decoding to implement parallel decoding algorithms on the Cell BE.

Shojania and Li were the first to demonstrate the effectiveness of parallelization in network coding with their *Progressive Parallelized Network Coding* algorithms [28]. However, our previous research has identified inefficiencies and unbalancing in their work, particularly with respect to large coefficient matrix sizes and has proposed Dynamic Vertical Partitioning (DVP) algorithm [31]. We employ the DVP algorithm here for the Cell BE system, and suggest enhancements, which require a balanced workload implementation, across the heterogeneous multi-core processor.

**Figure 5.**
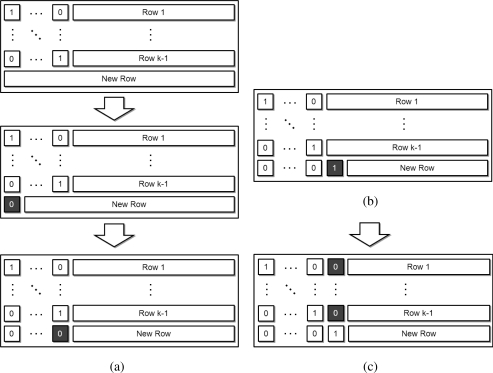
Processes on Stage A to Stage E; (**a**) During Stage A operation; (**b**) After Stage D operation; and (**c**) After Stage E operation.

Figure 5 presents the specific operations of progressive decoding, from *Stage A* to *Stage E* and Table 1, which introduced in [28] shows description of the operations and percentage of each operation step. In fact, Figure 5 depicts a decoding process after operations on the (*k* − 1)*_th_*’s row has just been finished and the *k_th_* row just arrives at the destination node.

Figure 5(a) depicts the operations at *Stage A*; the decoding process begins in the second figure within Figure 5(a). At the beginning, the first row is multiplied with the first element of the arriving row, and the resulting row is subtracted from the arriving row. The same operations are performed for the second row; it is multiplied with the second element of the arriving row and the resultant row is subtracted from the arriving row. These operations are continued until all leading values are reduced to “0”.

After the operations of *Stage A* are finished, the next decoding process identifies the first non-zero coefficient element (*Stage B*). It then determines whether the new row is linearly independent of the previously received rows (*Stage C*). The newly arriving row is then divided along the first non-zero element of the row, referred to as the *pivot* (*Stage D*) in Figure 5(b). Finally, we reduce the values of this same column across all previous rows to “0” (*Stage E*) depicted in Figure 5(c).

## 3. Load Distribution and Progressive Decoding on Cell BE

In the network coding research conducted previously by Shojania and Li [28], computational effort is statistically distributed amongst multiple threads. However, as the size of the coefficient matrix increases, dynamically distributed computation has the potential to improves the performance with well distributed load balancing as demonstrated in our previous research [31]. In this section, we first introduce the previously proposed three algorithms which are tested on the Cell BE system; in addition, we develop a new algorithm for using on the heterogeneous Cell BE processor considering the load balance.

**Figure 6.**
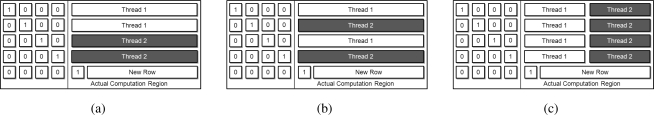
Parallelization algorithms of network coding on Homogeneous processor; (**a**) HP; (**b**) RRP; and (**c**) DVP.

In the previous work [31], three types of partitioning algorithms have been proposed, including Horizontal Partitioning (HP), Row by Row Partitioning (RRP), and Dynamic Vertical Partitioning (DVP); the three approaches are presented in Figure 6. In this figure, each algorithm reflects the relevant operation in *Stage E* when the fourth row is received and subsequently parallelized into two threads. Both HP and RRP divide the workload on a row-by-row bases. However, HP divides rows between threads in a sequential manner, while RRP divides them using a round-robin approach. DVP divides the workload with vertical and only takes the computational region into consideration. To implement these three algorithms on the Cell BE processor, we use SPEs to decode and the PPE to manage the SPE threads, to handle the synchronization, and to decode partial data which is distributed with considering load balancing between the asymmetric core properties.

### 3.1. Synchronization on the Cell BE

**Figure 7.**
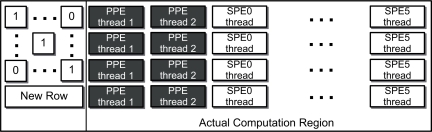
Dynamic resource distribution to Cell BE.

For an efficient decoding operation, we first distribute the computational region as shown in Figure 7. The Cell processor provided in Play Station 3, which is our experimental platform, is configured with two of the eight SPE cores disabled; therefore, we can only use seven programmable cores as one PPE core and six SPE cores. As the PPE has dual-threaded and dual issue hardware, it has two threads running simultaneously. Different from the PPE, the SPEs are able to manage only one thread per core. As indicated by the thread distribution method detailed in Figure 7, PPE *thread 1* manages pivot column’s elements and should transfer the elements to the other SPE threads before processing a newly received row. In addition, the Cell BE has a communication system called *mailbox* which delivers 32 bit data between the cores [32,33]. In fact, we use the mailbox system to synchronize the threads as well as to transfer the elements.

The mailbox system is designed for each SPE and implemented with an asymmetric manner; both the *inbound* and *outbound* mailboxes are contained in each SPE and messages are transmitted to the MFC from the SPE via the EIB. The mailboxes have one outbound entry and four inbound entries. At each SPE, a 32 bit inbound mail is read by the SPE and an outbound mail is sent by the SPE. A reading operation from SPEs stalls when the inbound mailbox entry is empty. As soon as a new message becomes available, the reading operation resumes. This stalling is also caused for a writing operation when the outbound mailbox entry is full.

Synchronization can be achieved by using the outbound mailbox entry in the following manner. Each SPE writes a mail in the outbound entry and continuously checks whether the PPE reads the mail and makes the outbound entry empty. At receiving all mails from the SPEs, the synchronization is achieved. This also implies that the PPE is responsible to wait until it receives all the mails. After the synchronization is guaranteed, each SPE waits a reply which contains a pivot element from the PPE.

On the other hand, we also propose to use the inbound mailbox solely for synchronization, which provides better performance with simple implementation. The PPE transfers the pivot element to the inbound mailbox entry and the SPE continuously checks until the pivot element is completely transferred. In this way, we can simply eliminate the necessity of synchronization messages from the SPE side. This is possible due to the fact that any stalled reading operation with an empty inbound entry can be used for the synchronization purpose.

### 3.2. Considering Load Balancing Effects on Cell BE

In this subsection, we propose our approach which enables an optimized workload distribution on the Cell BE. Figure 7 depicts the computational area required to process and to dynamically distribute the workload. The previous work has already considered load balancing on a general, homogeneous processor (e.g., Intel or AMD multi-core processors), however, the Cell BE is a heterogeneous processor which has an asymmetric core architecture. The SPEs have been designed to deliver higher computational power than the PPE, especially with the SIMD instruction set. As such, we must consider the difference between these two types of cores in order to achieve proper workload distribution.

For that purpose, we first have defined a value called *ppefactor* which decides the workload distribution ratio of the PPE *versus* the SPE. For example, when ppefactor is set to 0.1, the PPE takes 10% of the available work, and the remainder is assigned to the SPE. Before considering load balancing on the asymmetric core architecture, the cores would have equally divided workload. In order to find the optimal workload distribution, the proposed idea is strongly dependent upon heuristic.

Once the workload distribution over PPE and SPE is defined, the data partitioning to use the SIMD instructions should be defined. Although the data computation region is dynamically partitioned by DVP, architectural optimization can be achieved as the Cell BE processor supports those SIMD instruction set. The SIMD instructions for PPE and SPEs enables 128 bit operations. For that reason, the data are divided into chunks each of which is as large as 16 bytes. When the size of data is not a multiple of 16, the remainder is assigned to PPE. For example, When a data size is 117 bytes, each chunk is constructed from the right most element in the data (the right most column). This means that there exist 7 chunks and remaining 5 bytes which are the left most 5 bytes. Then, the five bytes are assigned to one of the PPE threads and remaining 7 bytes are assigned to the other PPE thread and SPEs. This method is superior to the method in which each core has an equal number of elements.

After addressing the workload distribution on each thread, we need to select a proper computation method between the table-based approach and the loop-based approach for Galois field multiplications. We now explain these two approaches in Section 3.3 and the selected method is then fully tested in Section 4.2.

### 3.3. Galois Field Operation for SIMD

The random linear network coding uses the Galois field numbers and accompanies computation overhead due to the time-consuming multiplication operations. In this subsection, we propose an optimization technique of Galois field operation which is previously proposed for GPU [34].

Increasing granularity of the Galois multiplication is hard to expand when using a table look-up method [34]. As the size of Galois field increases, memory requirement grows rapidly. In fact, increasing granularity of Galois field by 1 byte means a table size which is 256 times larger. This requires more cache and memory space. Furthermore, the SPEs do not have caches; they only have 256 KB SRAM, referred to as the local store. Therefore, it cannot contain any large sized tables or it can waste a large amount of local memory to hold the tables.

To provide sufficient granularity of the multiplications, Shojania *et al*. imported a loop-based approach which is based on the actual computations. Although the loop-based approach needs more computations than the table lookup method, it provides a faster computation time with the help of the SIMD instruction sets [28,34–36].

In the previous work [34], Shojania *et al*. suggested a word length wide multiplication method referred to as Rijndael’s finite field [37,38]. The method can perform four multiplication operations of the numbers in the Galois field at once. The Galois field numbers are as large as one byte and denoted as GF(2^8^).

**Figure 8.**
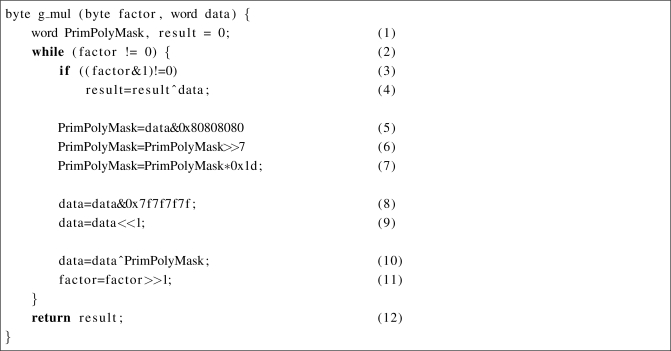
Optimized loop-based multiplication of GF(2^8^) for GPU.

They successfully applied the loop-based multiplication on the multiple scalar processors on a GPU which is depicted in Figure 8. The key optimization in the work is to eliminate branch operations by using polynomial mask operations. This helps to improve performance of a division operation with a irreducible polynomial variable. As a result, the execution time has been reduced.

Although they highly optimized the loop-based multiplication method by reducing diversity of control flow on branch instructions, there still exists possible reduction of one more branch instruction. Since the branch instruction causes stalls within a pipeline, any branch instruction in a loop crucially degrades performance of the Galois field multiplication; in fact, the speed of the Galois field multiplication highly affects the performance of network coding. For that purpose, we target to removes the remaining branch within the loop represented as (3) in Figure 8. This branch operation also can be replaced with the bitwise operations when the multiplication is optimized into the SIMD instructions. In addition, the replacement only causes less than five instructions to execute. On the other hand, if the branch instruction in line (2) is replaced to the bitwise operations, it requires a significant number of additional instructions to execute in the loop when the factor is zero. For that reason, the branch instruction in line (2) should remain for overall performance.

**Figure 9.**
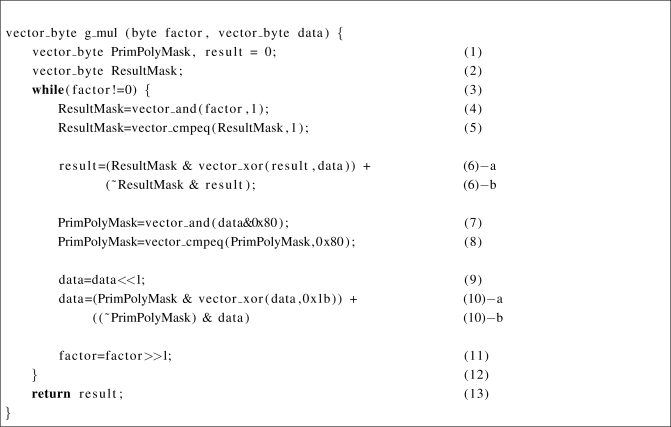
The loop-based SIMD multiplication in GF(2^8^).

The proposed Galois field multiplication based on the SIMD instruction set is shown in Figure 9. The main difference compared to the previous code in Figure 8 can be found in (4) to (6). The branch operation is replaced with the masking operation in *ResultMask* and the execution condition in the branch is calculated with a *vecor_cmpeq*, which is generally included in the SIMD instruction set. A *vector_cmpeq* operation checks whether each element in a vector is identical to the responsible element of the other vector. With comparing each element in both vectors, the operation set all bits of an element to 1 when the two elements are identical. Therefore, if the condition is true, the result becomes XOR-ed data (Figure 9(a)). Otherwise, the result is not changed (Figure 9(b)).

An SPE calculates 80 KB within 211 *μ*s with the original code. After the modification, an SPE finishes the same operation within 200 *μ*s. The optimization technique brings performance improvement 5.5%.

## 4. Experimental Results and Analysis

In this section, we first evaluate the previous parallelized network coding algorithm developed for the homogeneous multi-core processors on the Cell BE; we simply translate the previous approach to the SIMD instruction set of the Cell BE. Then, we compare the multiplication methods which are table-based, loop-based, and using SIMD instruction set multiplication. Further, we compare parallelized decoding performance of applying the specific multiplication methods on PPE and SPEs. We also evaluate partitioning of PPE workload applying the three multiplication methods adaptively, using *ppefactor*. Finally, we evaluate our parallelized progressive decoding method on the Cell BE and we compare it to the commercially available homogeneous multi-core systems, such as Intel and AMD quad-cores. The specifications of the evaluation environments are described in Table 2.

### 4.1. Implementation of Previous Work

**Figure 10.**
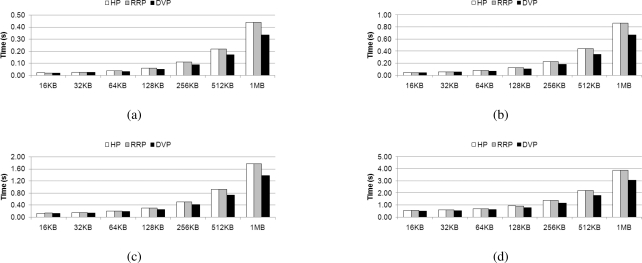
Decoding time of HP, RRP, and DVP on the Cell BE with various coefficient matrix size; (a) 64 × 64; (b) 128 × 128; (c) 256 × 256; and (d) 512 × 512.

We evaluate the previously proposed algorithms for homogeneous multi-core processors, HP, RRP, and DVP on the Cell BE architecture. Firstly, these algorithms are implemented with using only SPEs and SIMD instruction set for the SPE. Figure 10 presents execution time on the decoding operation that was discussed in Section 2.4. Experimenting with the entire coefficient matrix size, HP and RRP exhibit similar performance. In contrast, DVP exhibits even better performance. As the SPEs decode the data without a data cache, the dissimilarity between the HP and RRP algorithms does not affect the required decoding time.

The maximum performance difference between HP and RRP is only 1.69% and on average, there is only 0.04% difference. In addition, DVP shows a maximum 31% enhancement over HP and RRP. Therefore, in the next section, we perform the remaining experiments using DVP. As in the homogenous multi-core processor, the advantage of DVP in terms of load balancing brings better results. Detailed explanation on DVP can be found in [31].

The difference between Horizontal Partitioning (HP) and Row by Row Partitioning (RRP) comes from the different manner by which row is distributed to the different cores in *Stage E*. The results can be explained by the presence (or lack thereof) of a data cache. However, the Cell BE does not have a data cache on its SPEs. Therefore, there would be no distinctive difference between the two algorithms when implemented upon this architecture. In other words, the heterogeneous processor which has a simplified memory hierarchy to access local memory fast cannot provide efficiency of horizontal partitioning, even though it is a different and well balanced approach for cache embedded systems.

### 4.2. Computation Time on Galois Field

In this subsection, we evaluate the decoding performance of each Galois field multiplication method. For the analysis, we choose to use the 128 bit SIMD instruction set to parallelize the Galois field multiplications.

Let *COMPUTE* represent the loop-based algorithm, *TL* the table-based algorithm, and *VECTOR* the parallelized SIMD implementation of the loop-based algorithm, for the Galois field operations. We estimate the performance of the three multiplication methods on real machines: an Intel *Core 2 quad Q9400*, an AMD *Phenom-X4 9550*, and the Cell BE, all of which are described in Table 2.

**Figure 11.**
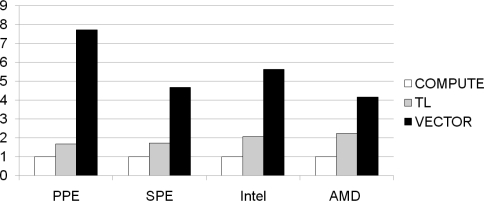
Speed-up of Galois Field operation.

Figure 11 presents the normalized performance of the *TL* and *VECTOR* methods over the *COMPUTE* method, on each type of core. All the cores display speed-up factors greater than ’1’ compared to the *COMPUTE* algorithm. In fact, *COMPUTE* obviously incurs greater overhead than *TL*, thus *TL* should be faster than *COMPUTE*. In addition, *VECTOR*, a parallelized method using SIMD instructions, is faster than all the other methods in processing 128 bit multiplications in parallel. The speed advantages in the PPE and SPE, obtained when using the *VECTOR* algorithm, are significant and noticeable.

In particular, the *VECTOR* algorithm executed on the PPE shows a speed increase by a factor of 7.71. Although the PPE incorporates data cache, just as other generic processors, the PPE has less than half of L1 data cache size compared to the other generic processors. In addition, the L2 cache is much smaller compared to the cache of the other general purpose processors. Therefore, the *VECTOR* algorithm exhibits a greater speed-up than other generic processors because it strongly depends on computation capability of SIMD execution unit. On the other hand, the SPE also has no data cache and merely has high-bandwidth embedded SRAM, referred to as the local store. However, it shows similar speed-up results compared to the other processors since its local store is as fast as the data caches.

**Figure 12.**
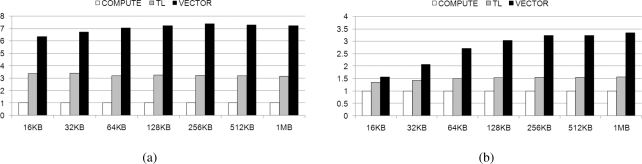
Speed-up of decoding time compared with COMPUTE on 128 × 128 coefficient matrix size; (**a**) PPE; (**b**) SPE.

In Figure 12, we present the speed increase exhibited by *TL* and *VECTOR* with respect to *COMPUTE*, in the performance of actual decoding, using each method on the Cell BE architecture. Each method using DVP (PPE with 2 threads and SPE with 6 threads) is evaluated on the different core architecture, with varying data sizes between 16 KB and 1 MB, on a coefficient matrix of size 128. In real decoding processes, the speed increase of *TL* is increased on PPE, but decreased on SPE compared with result on Figure 11. On the other hand, *VECTOR* shows lower performance. As *TL* depends on the performance of cache rather than computing power and performance of entire decoding process affected by cache, *TL* shows the improved performance with PPE. However, with the absence of data cache, SPE shows lower performance using *TL*. Furthermore, *VECTOR* requires computing power and entire decoding process has additional overhead compared to the single multiplication. Therefore, it represents lower speed-ups compared to the results shown on Figure 11.

Despite of the low performance on small data size of SPE, SPE represents similar speed-ups when data size becomes large. Since SPE should be controlled by PPE to synchronize the decoding process between SPEs and transfer data from main memory, the SPE shows lower performance with small data size when the synchronization and data transfer overhead charges large proportion.

From the results in Figure 12, we intuitively find the parallelized SIMD multiplication is the optimal solution to achieve high-performance decoding.

### 4.3. Synchronization with Mailbox System

**Figure 13.**
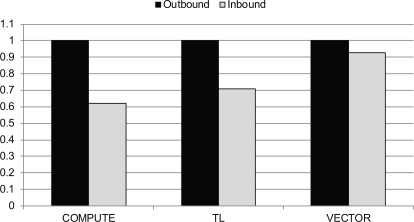
Inbound mailbox synchronization.

In Section 3.1, we introduce an efficient way to implement synchronization with the asymmetric mailbox system. With the inbound mailbox, the cores can synchronize at each decoding steps and can share values in the pivot column at once. We have compared decoding speed of the two synchronization methods based on inbound mailbox and outbound mailbox respectively in Figure 13. For the three kinds of computation approaches, COMPUTE, TL, and VECTOR, the decoding procedure is tested with varying the synchronization method and simply divided workload for each thread.

In experimental results, the synchronization method, which combines synchronization and the data transfer, reduces more than 10% of decoding time. COMPUTE and TL show remarkable reduced results since the three methods already have severe synchronization overhead by unfairness of workload distribution which does not consider the computation capability different types of cores. Consequently, the synchronization with inbound mailbox systems reduces performance degradation by inefficient synchronization methods and the performance degradation caused by absence of proper workload distribution. The performance improvement by well balanced workload is tested in the next subsection.

### 4.4. Partitioning on PPE

**Figure 14.**
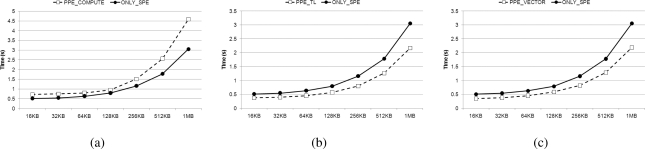
Decoding time of three algorithms which using PPE compared with only using SPEs with coefficient matrix size of 512; (**a**) *PPE_COMPUTE*; (**b**) *PPE_TL*; (**c**) *PPE_VECTOR*.

In Section 3.2, we explained the different factors that must be considered in determining workload distribution for the PPE and the SPEs. We have examined three multiplication methods on the PPE and compared result of each method to the performance achieved with utilizing only the SPEs. The performance results are depicted in Figure 13. The amount of workload dedicated on PPE is as large as the amount assigned to one SPE. We employ parallelized multiplication using the SIMD instruction set on the SPEs, rather than table-based multiplication, which is better suited to processors that have a local cache. Even if we also use the PPE in decoding, Figure 14 shows that lower increases in speed occur than are witnessed when only using the SPEs (with the exception of *PPE_VECTOR*).

In this section, we propose an approach to the factorization of workload between cores, and we evaluate the decoding time when varying distribution factor, which we refer to as *ppefactor*. Then, we use the configuration with equally divided workload distribution at each core as a performance baseline.

**Figure 15.**
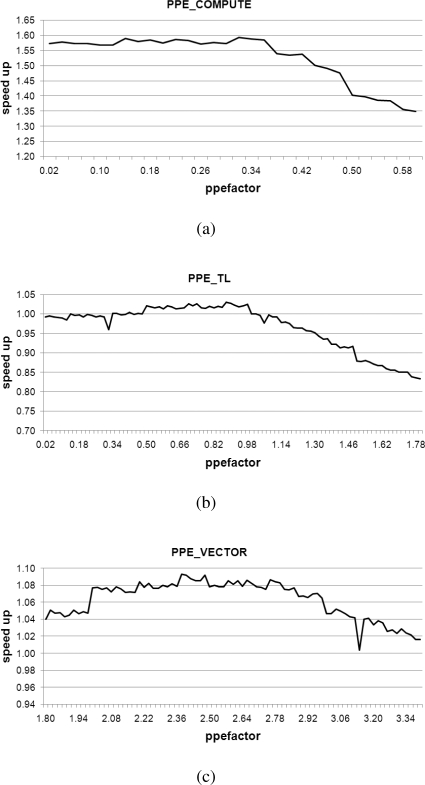
Speed-up with various ppefactor; (**a**) *PPE_COMPUTE*; (**b**) *PPE_TL*; and (**c**) *PPE_VECTOR.*

Figure 15 presents average speed-ups observed when varying the *ppefactor* for *PPE_COMPUTE*, *PPE_TL*, and *PPE_VECTOR* algorithms. *PPE_VECTOR* is a unified parallel algorithm that uses parallelized SIMD multiplication on either of the PPE and SPEs. In contrast, *PPE_COMPUTE* and *PPE_TL* are hybrid parallel algorithms. These employ computation-based and table-based multiplication on the PPE and they use only parallel SIMD multiplication on the SPEs.

In order to parallelize the progressive decoding algorithm across multiple cores, it is necessary to have a synchronization barrier that blocks excessive progression by any one particular thread. Synchronization employing a barrier greatly decreases performance when load balancing results in uneven distribution between cores. Thus, we evaluate the sensitivity of the three algorithms with respect to *ppefactor*. We do this because it will be necessary to dynamically redistribute the workload to all threads in an efficient manner, from a performance perspective.

**Figure 16.**
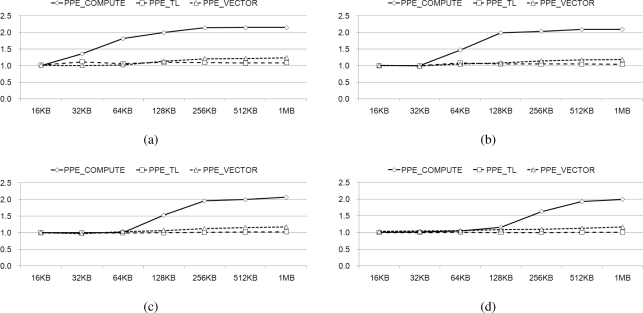
Speed-up of the algorithms compared with the result of having factor “1” when varying coefficient matrix size; (**a**) 64 × 64; (**b**) 128 × 128; (**c**) 256 × 256; and (**d**) 512 × 512.

Figure 16 depicts the measured average increase in speed that is observed when we decode data size from 16 KB to 1 MB, with a coefficient matrix varying in size from 64 to 512, with optimal values of *ppefactor*. For the small data size, since the portion that is assigned to PPE is smaller than the portion to SPE, its variation of the factor does not significantly affect the performance. However, if the data size is large enough to compare with coefficient matrix size then it shows high speed-up results.

In Figure 15(a–c), we can identify the most relevant local maximum values represented in Table 3, associated with each method. In Table 3, we have realized performance increase 8% with *ppefactor* of 2.38 even with *PPE_VECTOR*. It means that PPE is assigned a greater workload than the SPE. With this fine tuning on workload distribution, parallelization using the SIMD instruction set results in high performance on the Cell BE.

### 4.5. Overall Decoding Performance

We compare the performance results of our factorized parallelization, to the results obtained using a *ppefactor* of 1 in Figure 16. It presents comparison between the performance exhibited both before and after the factorization of the PPE, with varying sizes of the coefficient matrixes, from 64 to 512. After identifying the optimal *ppefactor*, we obtain a speed increase of more than 1.5, using *PPE_COMPUTE*. On the other hand, *PPE_VECTOR* and *PPE_TL* exhibit negligible speed increases. These results are arranged and presented in Table 3. It is readily apparent that factorization is an important consideration when we decompose and rearrange tasks on heterogeneous multi-core processors.

**Figure 17.**
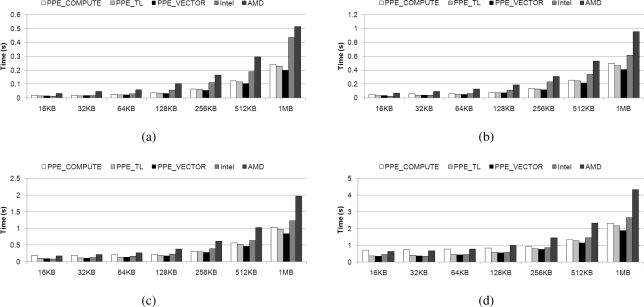
Decoding time on real machine with varying coefficient matrix size; (**a**) 64 × 64; (**b**) 128 × 128; (**c**) 256 × 256; and (**d**) 512 × 512.

Figure 17 presents observed decoding times with coefficient matrices of varying sizes, from 64 to 512, when decoding different volumes of data, between 16 KB and 1 MB. We have shown above, in Section 4.2, that the parallelized Galois field multiplications using the SIMD instruction is the fastest implementation method on a homogeneous multi-core processor. In order to ensure a legitimate comparison with our implementations on the Cell BE, we implemented network coding on the Intel and AMD quad-core processors using only SIMD instructions. We have compared computing-based (*PPE_COMPUTE*), table-based (*PPE_TL*), and SIMD-based (*PPE_VECTOR*) multiplication methods on the PPE to the SPE using SIMD-based multiplication. In addition, the implementations of *PPE_COMPUTE*, *PPE_TL*, and *PPE_VECTOR* exhibit average increases in speed of 0.32, 0.88, and 2.38, respectively, under experimental evaluation, as noted in Section 4.4. All implementations are compiled with the O3 level of the GNU GCC.

In Figure 17, it can be seen that *PPE_COMPUTE* demonstrates a low decoding speed when dealing with small data, however, it performs in a manner comparable to homogeneous processors as data size increases. This is because it incurs delay when the PPE is forced to wait for the SPEs during the decoding operation. In contrast, the other multiplication methods, which use table-based or parallelized SIMD-based multiplication on the PPE and parallelized SIMD-based multiplication on the SPE, on the Cell BE, exhibit fast decoding times in all experimental ranges. This gap increases with data size, as the gains from parallelization are enhanced.

**Figure 18.**
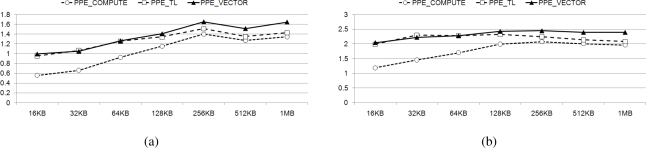
Average speed-up of network coding on real machine with varying data size; (a) Intel; and (b) AMD.

Figure 18 shows the average speed-ups varying the data size for all coefficient sizes; 64, 128, 256, and 512. It shows that the speed-ups are improved proportional to the data size; as the amount of computation increases, more data transmission to SPEs from main memory can be hidden. As we have shown in Figures 17 and 18, Cell BE is efficient for large data size of network coding when we use, especially, parallelized SIMD instruction.

## 5. Related Work

Ahlswede *et al*. were the first to introduce network coding and demonstrate its usefulness [1]. After this initial work, the maximum theoretical throughput of network coding was proven, and achieved, using linear network codes, by Koetter and Medard [39]. As suggested by Chou *et al*. [27] and Ho *et al*. [40], our implementations employ random linear network coding, which is believed to be the most practical approach to multicast flow scenarios, as the target to parallelize. Network coding research then spread to wireless network systems after its utility had been demonstrated by Lun *et al*. [41] in that context. Katti *et al*. proposed a number of practical solutions using multiple unicast flows [42] and Park *et al*. showed improvements in the reliability of ad hoc network systems [43].

The applications of network coding have been proposed in [44] and recent studies of feasibility in real testbeds have been performed and documented [45]. Especially, several previous literatures introduced to use network coding techniques in wireless sensor networks [5–10]. Widmer and Le Boudec introduced a network coding based forwarding scheme for wireless sensor networks where nodes sleep most of the time [5]. Al-Kofahi and Kamal handle the problem of survivability of many-to-one flows in wireless sensor networks (WSN) using the network coding technique [6]. In addition, Hou *et al*. proposed AdapCode, which is a reliable data dissemination protocol developed for any software update. Their proposed method relies on adaptive network coding to reduce broadcast traffics in the process of dissemination [9]. Using network coding in the design of practical health care wireless sensor networks is also presented in [10]. Using multi-core processors in the cloud computing environment also has been proposed [46].

In addition, Lee *et al*. introduced a discussion of the utility of network coding in mobile systems [47]. Further, Gkantsidis *et al*. showed that smooth, fast downloads and efficient server utilization can be achieved using network coding [4]. Lastly, Shojania and Li consider adoption the network coding to practical applications in mobile networks with the Apple iPhone [48].

Parallelized network coding was first suggested by Shojania and Li [28]. The authors used hardware acceleration and proposed a multi-threaded design utilizing multi-core systems. Research has also been conducted, from a variety of perspectives, which focuses on reducing the computational complexity of encoding/decoding operations [49,50]. Park *et al*. suggested enhanced forms of parallelization network coding algorithms with reduced computational complexity [31,51]. Whereas, our work is focused on improving decoding performance via the adoption of algorithms for use in a heterogeneous processor, referred to as the Cell BE.

Many algorithms have been proposed to parallelize matrix calculation, such as the parallelization of matrix inversion [52], parallel LU decomposition [29], and parallelization of Gauss-Jordan elimination with block-based algorithms [30]. However, due to the network transfer delay, Park *et al*. employ a more aggressive method of network coding, referred to as *“progressive”* decoding [28].

Approaches to enhancing the performance of the progressive decoding were proposed in *Parallelized Progressive Network Coding* [28]. The approaches are based on Gauss–Jordan elimination algorithm. A simple description of one variant of Gauss–Jordan elimination, as explained in [28], is presented in Table 1 of this paper. Over the entire decoding process, *Stage A* and *E* comprise the majority of the workload; according to [28], *Stage A* makes up 50.05% of the workload, while *Stage E* has 49.5%.

The load-balancing problem has been emphasized in divisible load theory [21–23]. Drozdowski and Lawenda introduced a method of verifying divisible load size for heterogeneous distributed systems [53]. Cariño *et al*. suggested a factoring method for dynamical load-balancing in [54]. The usefulness of hardware acceleration has been shown by Shojania *et al*. [34] and Chu *et al*. [55] on a GPGPU.

## 6. Conclusions

**Figure 19.**
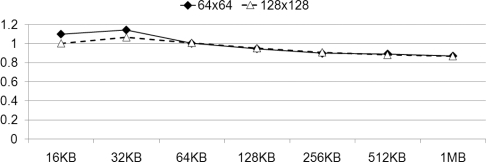
Speed-up of *PPE_TL* over *PPE_VECTOR* with varying data size.

In this paper, we introduced an efficient random linear network coding algorithm with an appropriate load balancing method for a heterogeneous multi-core processor. We especially designed the proposed architecture considering the wireless sensor network environment. Our algorithm introduced a proper load balancing method and a hybrid progressive decoding algorithm considering different computing capability of cores. We achieve a maximum speed increase by selectively using multiplication algorithms that are (1) table-based in dealing with small coefficient and data sizes and (2) parallelized and employing SIMD instructions in dealing with large coefficient sizes as shown in Figure 19.

We compared performance of the proposed approach to one of the fastest progressive decoding algorithms, executed on homogeneous processors. From this comparison, we demonstrated improved performance results using our method. Table 4 represents maximum and average speed-ups of network coding about various matrix sizes (64, 128, 256, and 512) compared to the homogeneous processors. Our proposed implementation shows improved performance in most of the experiments. We achieved a maximum speed-up of 2.19 at 1 MB data with a coefficient matrix of 64 compared to the Intel quad-core processor. In addition, we obtained a maximum speed-up of 3.12 at 128 KB data with coefficient matrix of 64 compared to the AMD quad-core processor. The proposed method shows greater efficiency in dealing with especially large data sizes.

## Figures and Tables

**Table 1.** Five Stages of Progressive Decoding [28].

**Stage**	**Procedure Description and Workload Distribution**	
**A**	Using the previous coefficient rows, reduce the leading coefficients in the new row to zero	**(50.05%)**
**B**	Find the first non-zero coefficient in the new coefficient row.	**(0.05%)**
**C**	Check for linear independence with existing coefficient rows.	**(0.00001%)**
**D**	Reduce the leading non-zero entry of the new row to 1.	**(0.38%)**
**E**	Reduce the coefficient matrix to the reduced row-echelon form.	**(49.5%)**

**Table 2.** Experimental Environments.

		**Sony PlayStation3**	**Intel Quad Core**	**AMD Quad Core**
	**CPU**	Cell BE	Intel Core 2 quad Q9400	AMD phenom-X4 9550
	**Clock**	3.2 GHz	2.66 GHz	2.2 GHz
	**RAM**	512 MB	2 GB	4 GB
**SPEC**	**Cache**	L1 : 32 KB	L1 : 4 × 64 KB	L1 : 4 × 128 KB
	**Size**	L2 : 512 KB	L2 : 2 × 3 MB	L2 : 4 × 512 KB
				L3 : 2 MB shared
	**OS**	Linux	Linux	Linux
		Yellow Dog Linux 6.1	Fedora Core7	Fedora Core8
	**Number of Cores**	(1 + 6)	4	4

**Table 3.** Speed-up compared Equally Distributed Decoding.

	**COMPUTE**	**TL**	**VECTOR**
**Optimal factor**	0.32	0.88	2.38
**Maximum speed-up**	2.15	1.42	1.26
**Average speed-up**	1.59	1.03	1.08

**Table 4.** Comparison of Homogeneous Processors.

		**COMPUTE**	**TL**	**VECTOR**
**Intel**	**Maximum speed-up**	1.80	1.90	2.19
	**Average speed-up**	1.05	1.27	1.36
**AMD**	**Maximum speed-up**	2.71	3.00	3.12
	**Average speed-up**	1.77	2.19	2.31

## References

[1] Ahlswede R, Ning C, Li S-YR, Yeung RW (2000). Network information flow. IEEE Trans. Inf. Theory.

[2] Sanchez-Avila C, Sanchez-Reillol R The Rijndael Block Cipher (AES proposal): A comparison with DES.

[3] Li B, Wu Y (2011). Network coding. Proc. IEEE.

[4] Gkantsidis C, Miller J, Rodriguez P Comprehensive view of a live network coding P2P system.

[5] Widmer J, Le Boudec JY Network coding for efficient communication in extreme networks.

[6] Al-Kofahi OM, Kamal AE (2009). Network coding-based protection of many-to-one wireless flows. IEEE J. Sel. Areas Commun.

[7] Woldegebreal DH, Karl H Network-coding-based cooperative transmission in wireless sensor networks: Diversity-multiplexing tradeoff and coverage area extension.

[8] Platz D, Woldegebreal DH, Karl H Random network coding in wireless sensor networks: Energy efficiency via cross-layer approach.

[9] Hou IH, Tsai YE, Abdelzaher T, Gupta I AdapCode: Adaptive network coding for code updates in wireless sensor networks.

[10] Egbogah EE, Fapojuwo AO (2011). A survey of system architecture requirements for health care-based wireless sensor networks. Sensors.

[11] Wu Y, Chou PA, Kung SY (2004). Information Exchange in Wireless Networks with Network Coding and Physical-Layer Broadcast.

[12] Widmer J, Fragouli C, LeBoude JY Energy efficient broadcasting in wireless *ad hoc* networks.

[13] Fragouli C, Widmer J, Le Boudec JY A network coding approach to energy efficient broadcasting: From theory to practice.

[14] Cai N, Yeung R Secure network coding.

[15] Cai N, Yeung R (2011). Secure network coding on a wiretap network. IEEE Trans. Inf. Theory.

[16] Liu Z, Wu C, Li B, Zhao S UUSee: Large-Scale operational on-demand streaming with random network coding.

[17] Geer D (2005). Chip makers turn to multicore processors. Computer.

[18] Ohara S, Suzuki M, Saruwatari S, Morikawa H A prototype of a multi-core wireless sensor node for reducing power consumption.

[19] Spies C, Indrusiak L, Glesner M Comparative analysis of multitask scheduling algorithms for reconfigurable computing regarding context switches and configuration cache usage.

[20] Akyildiz IF, Melodia T, Chowdhury KR (2008). Wireless multimedia sensor Networks: Applications and testbeds. Proc. IEEE.

[21] Bharadwaj V, Robertazzi TG, Ghose D (1996). Scheduling Divisible Loads in Parallel and Distributed Systems.

[22] Bharadwaj V, Ghose D (2003). Divisibleload theory: A new paradigm for load scheduling in distributed systems. Clust. Comput.

[23] Drozdowski M (1997). Selected Problems of Scheduling Tasks in Multiprocessor Computer Systems.

[24] Intel Microprocessor Export Compliance Metrics. http://www.intel.com/support/processors/sb/cs-023143.htm.

[25] Kahle JA, Day MN, Hofstee HP, Johns CR, Maeurer TR, Shippy D (2005). Introduction to the cell multiprocessor. IBM J. Res. Dev.

[26] Pham D, Aipperspach T, Boerstler D, Bolliger M, Chaudhry R, Cox D, Harvey P, Harvey P, Hofstee H, Johns C, Kahle J, Kameyama A, Keaty J, Masubuchi Y, Pham M, Pille J, Posluszny S, Riley M, Stasiak D, Suzuoki M, Takahashi O, Warnock J, Weitzel S, Wendel D, Yazawa K (2006). Overview of the architecture, circuit design, and physical implementation of a first-generation cell processor. IEEE J. Solid-State Circuits.

[27] Chou PA, Wu Y, Jain K Practical network coding.

[28] Shojania H, Li B Parallelized progressive network coding with hardware acceleration.

[29] Bisseling RH, van de Vorst JGG Parallel LU decomposition on a transputer network.

[30] Melab N, Talbi EG, Petiton S (2000). A parallel adaptive Gauss-Jordan algorithm. J. Supercomput.

[31] Park K, Park JS, Ro WW (2010). On improving parallelized network coding with dynamic partitioning. IEEE Trans. Parallel Distrib. Syst.

[32] Arevalo A, Matinata RM, Pandian MR, Peri E, Ruby K, Thomas F, Almond C (2008). Programming the Cell Broadband Engine Architecture: Examples and Best Practices.

[33] PPU & SPU C/C++ Language Extension Specification. https://www-01.ibm.com/chips/techlib/techlib.nsf/techdocs/30B3520C93F437AB87257060006FFE5E.

[34] Shojania H, Li B, Wang X Nuclei: GPU-Accelerated many-core network coding.

[35] AltiVec Technology Programming Interface Manual. http://www.freescale.com/files/32bit/doc/refmanual/ALTIVECPIM.pdf.

[36] (2010). Intel(R) 64 and IA-32 Architectures Optimization Reference Manual.

[37] Roth R (2006). Introduction to Coding Theory.

[38] Trenholme S AES’ Galois field. http://www.samiam.org/galois.html.

[39] Koetter R, Médard M (2003). An algebraic approach to network coding. IEEE/ACM Trans. Netw.

[40] Ho T, Medard M, Koetter R, Karger D, Effros M, Shi J, Leong B (2006). A random linear network coding approach to multicast. IEEE Trans. Inf. Theory.

[41] Lun D, Ratnakar N, Medard M, Koetter R, Karger D, Ho T, Ahmed E, Zhao F (2006). Minimum-cost multicast over coded packet networks. IEEE Trans. Inf. Theory.

[42] Katti S, Rahul H, Hu W, Katabi D, Medard M, Crowcroft J (2008). XORs in the air: Practical wireless network coding. IEEE/ACM Trans. Netw.

[43] Park JS, Gerla M, Lun D, Yi Y, Medard M (2006). Codecast: A network-coding-based *ad hoc* multicast protocol. IEEE Wirel. Commun.

[44] Gkantsidis C, Rodriguez P Network coding for large scale content distribution.

[45] Wang M, Li B Lava: A reality check of network coding in peer-to-peer live streaming.

[46] Kang M, Kang DI, Crago SP, Park GL, Lee J (2011). Design and development of a run-time monitor for multi-core architectures in cloud computing. Sensors.

[47] Lee U, Park JS, Yeh J, Pau G, Gerla M Code torrent: Content distribution using network coding in VANET.

[48] Shojania H, Li B Random network coding on the iPhone: Fact or fiction?.

[49] Lee U, Park JS, Yeh J, Pau G, Gerla M A content distribution system based on sparse linear network coding.

[50] Maymounkov P, Harvey NJA Methods for efficient network coding.

[51] Park K, Park JS, Ro WW Efficient parallelized network coding for P2P file sharing applications.

[52] Csanky L Fast parallel matrix inversion algorithms.

[53] Drozdowski M, Lawenda M (2007). Multi-installment divisible load processing in heterogeneous distributed systems: Research articles. Concurr. Comput. Pract. Exp.

[54] Cariño RL, Banicescu I (2008). Dynamic load balancing with adaptive factoring methods in scientific applications. J. Supercomput.

[55] Chu X, Zhao K, Wang M (2009). Accelerating network coding on many-core GPUs and multi-core CPUs. J. Commun.

